# A common mechanism involving the TORC1 pathway can lead to amphotericin B-persistence in biofilm and planktonic *Saccharomyces cerevisiae* populations

**DOI:** 10.1038/srep21874

**Published:** 2016-02-23

**Authors:** Rasmus Bojsen, Birgitte Regenberg, David Gresham, Anders Folkesson

**Affiliations:** 1 National Veterinary Institute, Technical University of Denmark, Frederiksberg, Denmark; 2 Department of Biology, University of Copenhagen, Copenhagen, Denmark; 3 Department of Biology, Center for Genomics and Systems Biology, New York University, New York, USA

## Abstract

Fungal infections are an increasing clinical problem. Decreased treatment effectiveness is associated with biofilm formation and drug recalcitrance is thought to be biofilm specific. However, no systematic investigations have tested whether resistance mechanisms are shared between biofilm and planktonic populations. We performed multiplexed barcode sequencing (Bar-seq) screening of a pooled collection of gene-deletion mutants cultivated as biofilm and planktonic cells. Screening for resistance to the ergosterol-targeting fungicide amphotericin B (AmB) revealed that the two growth modes had significant overlap in AmB-persistent mutants. Mutants defective in sterol metabolism, ribosome biosynthesis, and the TORC1 and Ras pathways showed increased persistence when treated with AmB. The *ras1*, *ras2* and *tor1* mutants had a high-persister phenotype similar to wild-type biofilm and planktonic cells exposed to the TORC1 pathway inhibitor rapamycin. Inhibition of TORC1 with rapamycin also increased the proportion of persisters in *Candida albicans* and *Candida glabrata.* We propose that decreased TORC1-mediated induction of ribosome biosynthesis via Ras can lead to formation of AmB-persister cells regardless of whether the cells are in planktonic or biofilm growth mode. Identification of common pathways leading to growth mode-independent persister formation is important for developing novel strategies for treating fungal infections.

Advances in medical procedures have increased the use of invasive devices and immunosuppressive treatments. This has led to increased numbers of patients susceptible to fungal infections^1^. The biomaterial of medical implants is suitable for fungal cell attachment and use of invasive devices is a risk factor for fungal biofilm infections^2^. Biofilm cells can survive high doses of antimicrobial agents and only echinocandins and polyenes have *in vitro* antibiofilm activity^3^,^4^. The polyene drug amphotericin B (AmB) targets ergosterol in the cell membrane and forms pores that rapidly lyse cells^5^. These fungicidal properties and broad spectrum of activity have made AmB the preferred agent for treatment of severe mycosis since its introduction in the late 1950s.

Clinical reports of fungal resistance to AmB are rare and known resistance mechanisms are limited to alterations in cell wall or sterol membrane patterns^5^,^6^. Nonetheless, biofilms are thought to become tolerant to AmB by sequestering the drug in the extracellular matrix^7^,^8^,^9^, decreasing membrane ergosterol levels^10^,^11^, or forming persister cells^12^. Persister cells remain viable after treatment with high doses of antimicrobial agents without heritable genetic changes. A persister subpopulation is typically about 1% of a population and consists of phenotypically tolerant variants of wild-type genotype. Once antimicrobial pressure is removed, this subpopulation can repopulate the infection site^13^. The clinical relevance of persister cells has been demonstrated in patients with oral candidiasis who receive antimicrobial therapy that selects for high-persister (*hip*) mutants. Persister mutants with *hip* phenotypes have minimal inhibitory concentrations (MICs) similar to wild-type, but generate a higher proportion of tolerant cells. Antifungal recalcitrance mediated by persister cells is a survival mechanism that might contribute significantly to treatment failure^13^, but cannot be detected by standard laboratory susceptibility tests. The mechanisms of persister formation in bacteria are well studied and involve toxin-antitoxin systems that inhibit protein synthesis and result in cellular dormancy^13^. Less is known about persistence in yeast, although histone deacetylases^14^ and superoxide dismutases^15^ are suggested to be involved.

Although biofilms and stationary phase planktonic populations share phenotypic properties including low metabolic activity, phenotypic heterogeneity, increased stress tolerance and persister formation^16^,^17^,^18^,^19^, biofilm research has mainly focused on differences between the two growth modes. Studies of tolerance mechanisms shared by planktonic and biofilm-forming cells could lead to discovery of novel treatment strategies that function independent of growth mode. One approach to characterizing general AmB-persister mechanisms is identifying mutants that have *hip* phenotypes under different growth modes. *Saccharomyces cerevisiae* is an experimental model for fungal biofilm studies^20^,^21^,^22^ and we have previously observed AmB-tolerant persisters in *S. cerevisiae* biofilm and planktonic populations^17^. Comprehensive barcoded gene-deletion strain collections are available for *S. cerevisiae* that enable the systematic study of protein function and genotype-to-phenotype correlations^21^,^23^,^24^. The unique barcode tags of each mutant and next generation sequencing facilitate multiplexed barcode-sequencing (Bar-seq) for high-throughput screens of pooled mutants^25^,^26^. To identify growth mode-independent persister mechanisms, we performed a genome-wide Bar-seq analysis of AmB-tolerance in *S. cerevisiae* biofilm and planktonic populations using a gene deletion collection in a biofilm competent strain^21^.

We found significant overlap in AmB-tolerance mutants between biofilm- and planktonic-growing cells, and many mutants uniquely identified in either growth mode had lost functions in metabolic and proliferative processes found to be important for tolerance in both growth modes. Several mutants were *hip*-phenotype mutants characterized by wild type minimal inhibitory concentration (MIC) values but with an increased subpopulation of AmB-persistent cells relative to wildtype. Many of these mutants were impaired in the regulation of growth and translation, suggesting a common mechanism underlying persister formation in planktonic growth and biofilms. In summary, we used genome-wide multiplexed Bar-seq analysis to identify *hip-*phenotype yeast mutants to understand how persistence arises in microbial populations. Our genetic analysis suggests that one mechanism leading to persistence is mediated by a general down-regulation of protein translation controlled by the TORC1 and Ras pathway activities.

## Results

### AmB-tolerant cells have a persister phenotype

**Figure 1:**
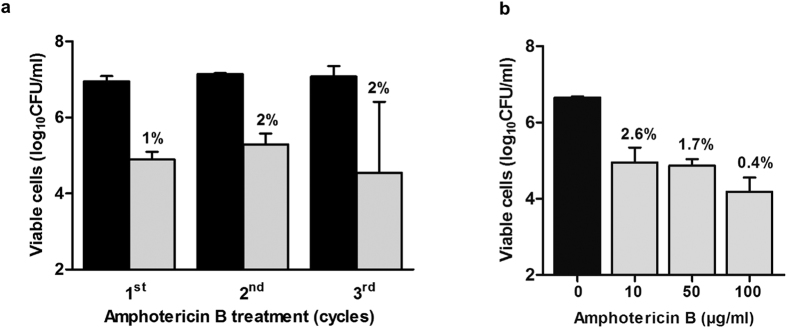
AmB persistence is not heritable. (**a**) 48-hour wild-type biofilms treated with 10 μg/ml amphotericin B (AmB) for 24 hours (gray) or untreated (black). Viability was measured as colony-forming units (CFUs). Surviving populations were reinoculated to form new biofilms that were re-exposed to AmB for three cycles. (**b**) Survival of 48-hour wild type biofilms treated for 24 hours with 10, 50, or 100 μg/ml AmB. n = 3, error bars show standard deviation.

Biofilms formed by *C. albicans*, survive AmB treatment by the formation of persister cells that are characterized by noninherited resistance^12^. We investigated if AmB resistance was also noninherited in our *S. cerevisiae* biofilm model by treating 24-hour biofilms with AmB and re-treating new biofilms formed by persisters with AmB for a total of three treatment cycles (Fig. 1a). The first cycle killed most cells, with 1% surviving AmB treatment. The biofilm populations produced by the surviving cells showed similar AmB tolerance, with 2% viability. No significant differences in the sizes of the AmB-tolerant populations were observed during the cycles (P = 0.7314, one-way ANOVA), suggesting that the survival mechanism was not heritable. Since persister cells survive treatment with high concentrations of antifungal agents, we determined the correlation between drug concentration and cell viability of the AmB-tolerant subpopulation. Exposing biofilm cells to 10 μg/ml AmB for 24 hours (Fig. 1b) resulted in survival of 2.6% of the population. Exposure to 50 μg/ml led to survival of 1.7% and treatment with 100 μg/ml resulted in survival of 0.4%. Although there was a trend towards fewer persisters with increasing AmB treatment, it was not statistically significant (P = 0.0517, one-way ANOVA).

### Screening for AmB-tolerant *hip* mutant strains using multiplexed barcode sequencing

We used Bar-seq to identify genes that contributed to the generation of AmB-tolerant persister cells in biofilm and planktonic populations. A barcoded mutant collection was pooled and four biological-replicate cultures were distributed to plastic surfaces for biofilm growth or glass flasks for planktonic growth. After 4 days, biofilm and planktonic cells were treated with AmB or left untreated. After another 12 hours, treated and untreated cell samples were outgrown for 24 hours to enrich for surviving mutants before barcode sequencing (Supplementary Fig. S1). Differential mutant abundance after AmB treatment was analyzed using the Bar-seq analysis pipeline developed by Robinson *et al.*^27^.

**Figure 2:**
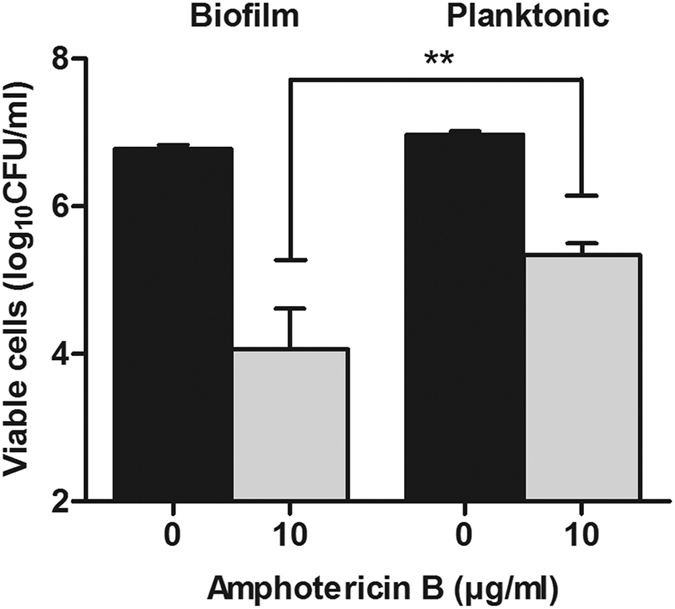
Barcode sequencing screen. Number of viable cells in the pooled mutant collection measured as colony-forming units (CFUs) of untreated control (black bars) and amphotericin B-treated cells (grey bars). n = 4, error bars show standard deviation. Statistical significance between AmB treated samples of biofilm and planktonic cells was evaluated with Student’s *t*-test. **P < 0.005.

Dimensionality reduction of the mutant survival data showed that all biological replicates for the untreated control samples grouped together and untreated control samples for biofilm and planktonic growth were in the same cluster (Supplementary Fig. S2). This result indicated high similarity in the abundance of mutants in the biofilm and planktonically cultivated populations. For AmB-treated samples, three of four biological replicates were grouped for both the biofilm and planktonic grown cells. After treatment of the pooled biofilm population with AmB, the mean viable population decreased to 0.3% of initial population size (Fig. 2). AmB treatment of the planktonic population resulted in mean survival of 2% of initial population size. Hence, the tolerant population was approximately 10 times larger after planktonic growth compared to biofilm growth showing a higher proportion of tolerant cells among the planktonically grown cells (P = 0.0043, Student’s *t*-test).

### Mutants affected in lipid metabolism, protein synthesis and cell cycle genes increase AmB tolerance

Bar-seq analysis identified 69 mutants with significant (FDR < 0.005) differential abundance in biofilm cells after AmB treatment and 121 in planktonic cells (Supplementary Fig. S3 and Supplementary Table S1). Of these, 32 mutants were shared between the two growth modes representing a significant overlap (P < 0.0001, two-tailed Fisher’s exact test). No gene ontologies were significantly overrepresented among the mutants. However, following manual curation using GO Term Finder in SGD^28^ (Supplementary Table S2) mutants enriched in both biofilm and planktonic populations indicated similar persistence mechanisms in biofilm and planktonic cells (Table 1).

AmB binds to cell membrane ergosterol that result in pore formation and rapid cell lysis^5^. Thus it was expected to find mutants in the sterol biosynthesis genes in our screen. *cyb5* and *erg5* increased AmB survival in biofilm and planktonically cultivated cells, while mutation of two ergosterol biosynthesis genes, *NCP1* and *ECM22*, conferred resistance in planktonic cells only (Table 1). Deletion of these genes likely resulted in structural modifications of sterols leading to reduced binding of AmB to the plasma membrane. Structural modification of ergosterol is a known resistance mechanism against AmB^5^,^29^ and the identification of ergosterol mutants positively validated the Bar-seq screen. In addition, deletion of the sterol transporter gene *OSH2* and the lipid homeostasis regulator gene *SAH1* increased AmB survival in both growth modes (Table 1). Sterol involvement in AmB survival was further supported by increased abundance of the sterol homeostasis mutant *tgl1* in biofilm-cultivated cells. Identification of enriched *cki1* in biofilm cells, *inp51* in planktonic cells and *sac1* in both cell types suggested involvement of phosphatidylcholine and sphingolipid membrane lipids in AmB sensitivity.

A large group of mutants had defects in genes involved in cell cycle progression, protein synthesis and protein turnover; we identified several ribosomal proteins and ubiquitination-associated genes in both biofilm and planktonic cells. Loss of the protein kinases Elm1 and Gin4, which are involved in novel daughter cell growth and septin ring formation^30^, led to enrichment in both planktonic and biofilm populations. Enrichment of *bud9*, *gic1*, *hsl7* in biofilm and *pcl9* in planktonic cultures after AmB treatment supported a role for bud growth and polarization in *S. cerevisiae* susceptibility to AmB.

### Mutants produce a high proportion of AmB-tolerant persister cells

**Figure 3:**
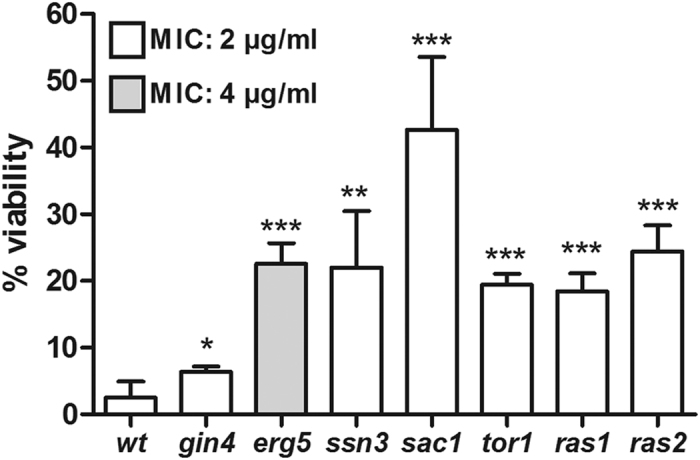
Persister cell levels in enriched amphotericin B-tolerant mutants. Viability was determined as colony-forming units (CFUs) after two hours exposure to amphotericin B. MIC, Minimal inhibitory concentration. wt, wild-type; n = 3–6; error bars show standard deviation. Statistical significance was evaluated with Student’s *t*-test. *P < 0.05, **P < 0.005, ***P < 0.0001.

To investigate if AmB-tolerant mutants possessed a *hip* phenotype, we compared MIC values and persister cell levels in individually cultured mutants and wild-type. We included *ssn3* (cyclin-dependent regulation of RNA polymerase II), *erg5* (ergosterol biosynthesis) and *sac1* (phosphatidylinositol dephosphorylation), which were the most significantly differentially abundant mutants with known biological processes and were AmB persistent in both biofilm and planktonic cultures. We included *gin4* (bud growth and assembly of the septin ring) to represent the large group of mutants affected in cell cycle progression. One of the characteristics of *hip* mutants is that they have wild-type MIC values. Consistent with a *hip* phenotype, MIC determinations showed that only *erg5* had reduced AmB susceptibility whereas all other tested mutants had MIC values similar to wild-type (Fig. 3). Because the MIC assay measures the ability to grow in the presence of an antifungal agent, these results suggested that the high level of AmB tolerance of the mutants could be attributed to a nongrowing state similar to persister cells.

To investigate AmB persister cell levels in the mutants, we treated *S. cerevisiae* with AmB and determined viability before and after treatment. Treatment of all tested mutants significantly increased the surviving subpopulations (Fig. 3) from 3% in the wildtype to 6% in *gin4* (P = 0.0342, Student’s *t*-test), 23% in *erg5* (P < 0.0001), 22% in *ssn3* (P = 0.0009) and 43% in *sac1* (P < 0.0001). These results showed that, with the exception of *erg5,* the mutants were not directly resistant to AmB, but were enriched in the screen because they produced more persister cells than wild-type. We therefore considered them *hip* mutants.

### TORC1 and Ras pathways are involved in AmB tolerance

One group of mutants enriched in both planktonic and biofilm populations were deleted in the TORC1-activating EGO complex genes (*gtr1, gtr2, meh1,* and *slm4*)^31^,^32^ (Table 1). The highly conserved TORC1 pathway regulates cellular growth, is activated by nutrients, and inhibited by rapamycin^33^. The TORC1 pathway regulates cell cycle initiation and translation by transcriptional induction of ribosome protein genes^34^,^35^. The increased AmB tolerance in ribosomal protein and EGO mutants could indicate involvement of the TORC1 pathway in AmB tolerance for both biofilm and planktonic cells.

The *tor1* and *tor2* mutants were not represented in our Bar-seq screen because *tor2* is essential and *tor1* had low abundance and data from *tor1* were removed during data analysis. To investigate if TORC1 itself is involved in AmB sensitivity, we exposed *tor1* cells to AmB. The surviving mutant subpopulation was 19% and significantly (P < 0.0001, Student’s *t*-test) larger than the surviving wild-type population (Fig. 3). This increase was not caused by a change in MIC value (Fig. 3), suggesting a *hip* phenotype for *tor1*. TORC1 links nutrient sensing to induction of translation via the Ras pathway^35^. Among the 32 mutants with increased AmB tolerance in both biofilm and planktonic cultivated cells, the identification of *ras2* supported a potential connection between TORC1 and protein synthesis in AmB tolerance. Like the *tor1* mutant, the *ras2* culture did not have an altered MIC (Fig. 3) but showed a significantly (P < 0.0001, Student’s *t*-test) increased number of cells that survived AmB treatment (24%, Fig. 3). We also analyzed mutations in *RAS1*, a *RAS2* paralog, and found an AmB tolerance phenotype similar to *tor1* and *ras2* (Fig. 3).

**Figure 4:**
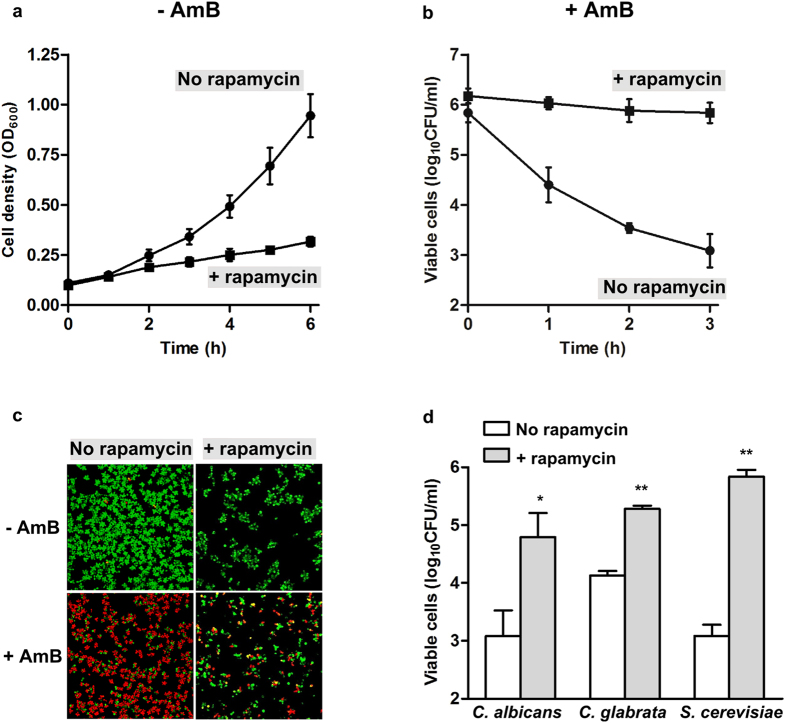
TORC1 inhibition mediates amphotericin B tolerance in wild-type planktonic and biofilm cultures. (**a**) Planktonic cells grown in YPD were treated with 1 μg/ml rapamycin (+rapamycin) or untreated (no rapamycin). Samples were extracted hourly for optical density measurements. n = 3, error bars show standard deviation. (**b**) Survival of exponential growing (No rapamycin) or rapamycin treated (+rapamycin, 1 μg/ml) planktonic cells exposed to 10 μg/ml amphotericin B (AmB). Viability was measured as colony-forming units (CFUs). n = 3, error bars show standard deviation. (**c**) Confocal laser scanning microscopy of AmB activity against four-hour biofilms after pre-exposure to 1 μg/ml rapamycin or no rapamycin. Survival is visualized with LIVE/DEAD staining. Green, live cells; red, dead cells. (**d**) Survival of exponential growing (No rapamycin) or rapamycin treated (+rapamycin, 1 μg/ml) planktonic cells exposed to five times MIC amphotericin B (AmB, 5 μg/ml against *Candida* spp. and 10 μg/ml against *S. cerevisiae*). Viability was measured as colony-forming units (CFUs) after 3 hours treatment. n = 3, error bars show standard deviation. Statistical significance between rapamycin treated and untreated samples was evaluated with Student’s *t*-test. *P < 0.05, **P < 0.001.

We investigated if growth arrest by chemical inhibition of TORC1 increased the proportion of AmB-tolerant persister cells. Rapid killing kinetics was observed after AmB treatment of exponentially growing cells resulting in killing of >99% of the cells after three hours (Fig. 4). Treatment of the wild-type with the TORC1-inhibiting agent rapamycin reduced growth (Fig. 4a) and resulted in slow AmB killing, with a ~500-fold increase in AmB tolerant cells compared to control (Fig. 4b). Rapamycin exposure also increased the proportion of biofilm cells that survived AmB treatment. Rapamycin-treated biofilm cells showed inhibited growth and a lower population density than untreated cells (Fig. 4c). Growth arrest of biofilm cells induced by rapamycin, followed by AmB treatment resulted in a higher proportion of surviving cells compared to cells not pre-exposed to rapamycin (Fig. 4c). This result supports our genetic results showing that inactivation of TORC1 in *S. cerevisiae* increased survival of both biofilm and planktonic cells after AmB treatment.

### Inhibition of TORC1 significantly increases the proportion of AmB persisters in *C. albicans* and *C. glabrata*

To validate if the results obtained in the model organism *S. cerevisiae* can be translated into human fungal pathogens, we inhibited TORC1 with rapamycin in *Candida albicans* and *Candida glabrata* and investigated the level of AmB persister cells. Figure 4d shows the number of viable cells after AmB treatment, and compares persister levels in TORC1 inhibited cells (+rapamycin) with exponential growing controls (No rapamycin). The results show that inhibition of TORC1 generates a significantly larger proportion of AmB tolerant persister cells in *C. albicans* (P = 0.0485, Student’s *t*-test) and *C. glabrata* (P = 0.0003) compared to controls, similar to *S. cerevisiae* (P < 0.0003).

## Discussion

Drug-tolerant persister cells survive high concentrations of antimicrobial agents while having MIC values similar to wild-type. Subpopulations of *S. cerevisiae* that tolerate AmB are phenotypically similar to AmB-tolerant persister cells in *Candida* biofilms^12^,^17^. Therefore, we performed a multiplexed Bar-seq screen using a *S. cerevisiae* gene deletion collection, to identify pathways and processes involved in AmB persistence in biofilm and planktonically cultivated cells. Our Bar-seq screen of *S. cerevisiae* led to identification of several mutants affected in (i) regulation of growth and in (ii) lipid metabolism, including several affected in ergosterol synthesis. Deletion of ergosterol synthesis genes likely resulted in decreased affinity of AmB to the plasma membrane, and AmB-tolerant persister cells might have lower levels of membrane ergosterol than susceptible cells. This conclusion is supported by the observation that *Candida* biofilms have decreased ergosterol levels^12^ and subpopulations of highly AmB-tolerant cells reduce transcription of ergosterol synthesis genes compared to the average biofilm^11^. The *S. cerevisiae* mutants that were enriched in the Bar-seq screen after AmB treatment had phenotypic properties similar to *C. albicans hip* mutants^36^: similar MIC value as the wild type, but a higher proportion of AmB-tolerant cells. *hip* mutants have been characterized in *C. albicans*, but the genetic basis for persistence is poorly understood.

We found that in *S. cerevisiae*, inhibition of the TORC1 complex, the TORC1-activating EGO complex and the TORC1-activated Ras pathway significantly increased the level of AmB tolerant cells, suggesting that reduced TORC1 and Ras activities were responsible for *S. cerevisiae hip* phenotypes. Ras signaling regulates programmed cell death in response to fungicidal exposure^37^,^38^, and persister cells might resist Ras-induced apoptosis that kills susceptible cells treated with antifungals^14^. In addition, *ras1* and *ras2* mutants have higher levels of AmB-tolerant persister cells than wild-type^37^, consistent with our results. Our finding of high AmB persistence in *ras1, ras2*, and *tor1,* and mutation of ribosomal protein genes suggests a general *hip* mechanism in *S. cerevisiae* caused by reduced ribosome synthesis through the TORC1-EGO-activated Ras pathway. TORC1 regulates ribosomal gene expression via Ras and the forkhead transcription factor Fhl1^35^, therefore, this is likely to be one pathway for AmB persistence. Other Ras pathway members were not identified in our screen because they are essential (Ifl2, Esa1, Rap1), are not in the mutant collection (Fhl1), or have several isoforms that activate pathway steps (Tpk1, Tpk2, Tpk3).

How the TORC1-EGO-activated Ras response causes AmB persistence is not evident from our current data. However, a plausible hypothesis is that quiescent cells arrested in G0 contain persistent subpopulations that have reduced ability to produce ribosomes or another TORC1/Ras-regulated response. Quiescent cells survive extended periods of starvation and resume proliferation when growth conditions become favorable^39^,^40^ comparable to the phenotypic properties of persister cells. We suggest that TORC1-dependent or Ras-dependent quiescence leads to AmB persistence and that this is a mechanism for persister formation common to biofilm and planktonic growth modes. These results validate the use of *S. cerevisiae* as model organism for studying antifungal resistance in *Candida* spp.

We found 158 gene deletions affected survival of yeast cells grown as biofilms or planktonically. The diversity of processes involved in AmB tolerance was similar to the range of mechanisms that generate antimicrobial persisters in bacteria, which is hypothesized to result from fluctuating variability in cellular processes^41^,^42^. Many of the genes we identified in the Bar-seq screen might not be directly involved in ergosterol syntesis, TORC1-mediated or Ras-mediated AmB resistance but affect other processes that impact cell cycle progression. For example, we also identified high levels of AmB-tolerant persister cells compared to wild-type in mutants with defects in small molecule metabolism and intracellular transport in addition to a large group of genes with unknown protein function. It is therefore important to note that yet undiscovered mechanisms might be equally important as TORC1 and Ras for generation of AmB persister cells.

We found that biofilm and planktonic populations share AmB-tolerance mechanisms and that a common *S. cerevisiae hip* response involves the TORC1 and Ras pathways. Although we identified 37 mutants uniquely involved in AmB tolerance in biofilm and 89 uniquely involved in planktonic cells, several mutants are affected in overlapping biological processes. A significant number of genes involved in AmB tolerance overlapped between the two growth modes and 17 of the 20 most significant contributors to tolerance in biofilm cells were also significant contributors to planktonic cell survival. This finding does not exclude specific biofilm AmB-tolerance mechanisms in *S. cerevisiae*, but shows the existence of common tolerance mechanisms between biofilm and planktonic populations, which has also been observed in bacteria^18^,^19^,^43^.

Diagnosing patients with biofilm infections can be difficult and laborious^44^. Therapies for microbial infections that are independent of growth mode and target both biofilm and planktonic cells could lead to faster patient treatment. However, growth-independent treatment initiatives require knowledge of common drug-tolerance mechanisms and shared targets between biofilm and planktonic cells. Our findings identified shared drug-tolerance mechanisms that could serve as a foundation for developing novel treatment strategies against fungal infections that are independent of growth mode. Our data suggest that inducing exit from TORC1-mediated quiescence could be a strategy to reverse the tolerance phenotype of persister cells, but further studies are needed to investigate this. Organ transplant recipients treated with rapamycin as an immunosuppressant agent have increased risk of being infected with fungi, which often requires antifungal drug therapy to eradicate the infection^45^,^46^. Furthermore, observations of *in vitro* synergistic activity of rapamycin in combination with AmB suggests a potential as novel drug combination against systemic fungal infections^47^,^48^. However, our results raise the possibility that the use of rapamycin in combination with conventional antifungal agents may increase the probability of generating persister cells.

## Materials and Methods

### Strains

*Saccharomyces cerevisiae* Σ1278b YS-11 (*MAT***a** *can1Δ*::*STE2p-spHIS5 lyp1Δ*::*STE3p-LEU2 his3*::*HisG leu2Δ ura3Δ*) was used as reference wild-type strain^21^. The *S. cerevisiae* Σ1278b haploid gene-deletion collection (*MAT***a** *can1Δ*::*STE2p-spHIS5 lyp1Δ*::*STE3p-LEU2 his3*::*HisG leu2Δ ura3Δ*)^21^ was transferred from 96-well glycerol stock plates to yeast extract peptone dextrose (YPD) agar and incubated at 30 °C for three days. Colonies were subsequently pooled in YPD medium and incubated overnight in glass flasks with agitation at 30 °C to obtain a mixed population of haploid mutants. *Candida albicans* ATCC 90028 and *Candida glabrata* ATCC 90030 were obtained from American Type Culture Collection.

### Media and growth conditions

All Bar-seq and all biofilm experiments were performed in minimal medium with 6.7 g/l yeast nitrogen base without amino acids (BD Difco) and 2.0 g/l glucose supplemented with amino acids (Supplementary Table S3). In all other experiments YPD was used to cultivate cells to verify the general applicability of our findings. All *S. cerevisiae* experiments were performed at 30 °C and all *Candida* spp. experiments were performed at 37 °C. AmB (A2411, Sigma) was dissolved in DMSO in 5 mg/ml stock solution and kept at −20 °C. Final DMSO concentrations in all assays were <1%^49^.

### Minimal inhibitory concentration (MIC)

MIC was determined as previously described^17^. In short, a two-fold dilution series of AmB was prepared in fresh medium and distributed into 96-well microtiter plates. Visibly turbid overnight cultures were diluted in fresh medium and transferred to the microtiter plates in OD_600_ 0.05 final cell densities. The microtiter plates were incubated with agitation (60 rpm) at 30 °C for 24 h. Biomass was measured with a microplate spectrophotometer (BioTek PowerWave 340). Growth inhibition of ≥90% was determined as MIC as recommended by EUCAST^50^. MIC values were determined in YPD medium and minimal medium with 2% glucose.

### Phenotypic characterization of drug-tolerant biofilm subpopulation

Yeast cultures at OD_600_ 0.1 were distributed into microtiter plates with a polystyrene surface. After 48 hours static incubation, medium with planktonic and loosely adherent cells was removed and centrifuged (2 min at 10,000 × *g*). Biofilm cells attached to the microtiter plate surfaces were challenged for 24 hours with the indicated concentrations of AmB in the spent medium. Biofilm cells were washed twice in saline and serially diluted in saline before plating on YPD agar. Inheritance of the drug-tolerant phenotype was investigated as previously described^12^ with modifications. In short, biofilm cells exposed to AmB at 10 times the MIC were suspended in fresh medium and transferred to a new microtiter plate. After two days, the biofilm cells were exposed to AmB at 10 times MIC. This process was repeated in a total of three re-inoculation and treatment cycles.

### Bar-seq screen

The pooled mutant collection was inoculated in four separate cultures of 700 ml minimal medium at a cell density of 10^5^ colony-forming units per ml (CFU/ml). After 5 hours with agitation at 30 °C, 50-ml samples were distributed in CELLSTAR Cell Culture Dishes (Greiner Bio) and sealed with parafilm to prevent evaporation for biofilm growth, or in glass flasks for planktonic growth. After 96 hours, the mean biofilm cell density was 6.1 × 10^6^ CFU/ml and the mean planktonic cell density was 9.4 × 10^6^ CFU/ml. AmB was added to the biofilm and planktonic populations at 10 μg/ml final concentration, corresponding to 10 times the MIC in minimal medium. AmB was added directly into planktonic cultures. For biofilm-growing cells, nonattached cells were removed from the medium by centrifugation (10 minutes at 4500 × *g*) and AmB was added to the spent medium to 10 μg/ml final concentration before reintroduction to biofilm cells. Drug-treated samples were protected from light with foil and incubated for 12 hours.

### DNA extraction from viable mutants

Biofilm-growing cells were harvested by removing media with nonadhering cells, and washing the remaining biofilm population in saline. Biofilm cells were scraped off the surface with a Drigalski spatula and resuspended in saline. Planktonic-growing cells were centrifuged for 10 minutes at 4500 × *g* and pellets were washed once in saline. One ml harvested cell culture was transferred to 25 ml YPD and outgrown at 30 °C with agitation to enrich for viable mutants. Genomic DNA was extracted with Wizard Genomic DNA Purification kits (A1125, Promega).

### Library preparation

Amplification of molecular barcodes from genomic DNA and incorporation of the *Illumina* adaptor sequences was performed in a two-step PCR protocol as previously described^26^ with a few modifications. In short, UPTAGs and DNTAGs were amplified in the first PCR reaction with unique index primers (Supplementary Table S4) and purified PCR concentrations were estimated from gel band intensities. UPTAGs and DNTAGs were combined in separate pools at 10 ng for each library. In a second PCR step, the *Illumina* adaptor sequence was incorporated into UPTAGs and DNTAGs in two separate PCR reactions. The two PCR products were combined in equimolar amounts and diluted to 2 nM final concentration. The library was sequenced in a single lane (HiSeq 2000, Illumina).

### Data analysis

Sequence reads were matched to a library of known barcode sequences using BarNone with default settings^25^. The HO knockout strain was present multiple times in the 96-well stock plates that the deletion collection was pooled from. This strain accounted for 30% of total sequence reads and was removed from the dataset. UPTAG and DNTAG reads (Supplementary Fig. S4) within each biological replicate were summed and normalized to total reads per sample. Low-abundance mutants present at fewer than 100 counts per million in fewer than four samples were filtered, leaving 2051 mutants for analysis. The dataset followed an overdispersed Poisson distribution (Supplementary Fig. S5). EdgeR (version 3.2.4)^27^ was used for differential abundance analysis as previously described^26^,^51^. False discovery rate (FDR) < 0.005 was considered significant. Dimensionality reduction analysis was generated by EdgeR and compares the relationship between all pairs of samples, using a count-specific pairwise distance measure. Distances correspond to leading log-fold change between each pair of sample^27^,^51^.

### Gene ontology

Genes significantly overrepresented after AmB treatment were manually assigned a biological process and grouped based on gene ontology (Supplementary Table S2) as annotated by the Saccharomyces Genome Database (SGD)^28^.

### Persister level determination

Exponentially growing cells were dissolved in fresh, preheated YPD medium to OD_600_ 0.1 in glass flasks. Cultures were incubated with agitation (200 rpm) and growth curves were performed by measuring optical density at absorbance 600 nm hourly. Exponentially growing cells were treated with five times the AmB MIC and viability was determined as CFUs. Rapamycin-induced target of rapamycin (TOR) inhibition was performed by exposing exponential growing populations to 1 μg/ml rapamycin (R0395, Sigma) prior to AmB treatment. AmB was added after four hours of rapamycin pre-exposure.

### Confocal microscopy of biofilm

Visibly turbid cultures were diluted to OD_600_ 0.1 and transferred to biofilm chambers (Technical University of Denmark^17^) with a polyvinyl chloride coverslip surface (Rinzl, Electron Microscopy Sciences) and cultivated for 4 hours before adding AmB for 3 hours. TORC1 inhibition of biofilm cells was by incubation of fresh cultures with 1 μg/ml rapamycin for 4 hours followed by 3 hours AmB treatment at 10 times the MIC. Staining for 15 minutes with 5 μM Syto9 (Invitrogen) was used to visualize live cells and 20 μM propidium iodide (Sigma-Aldrich) was used to stain dead cells. Confocal laser scanning microscopy (CLSM) was performed with a Zeiss LSM710 microscope equipped with excitation lasers at 488 nm and 514 nm. Imaging used an EC Plan-Neofluar 40x/1.30 oil lens.

## Additional Information

**How to cite this article**: Bojsen, R. *et al.* A common mechanism involving the TORC1 pathway can lead to amphotericin B-persistence in biofilm and planktonic *Saccharomyces cerevisiae* populations. *Sci. Rep.* **6**, 21874; doi: 10.1038/srep21874 (2016).

## Supplementary Material

Supplementary Information

Supplementary Table S1

Supplementary Table S2

## Figures and Tables

**Table 1:** Mutants enriched in the barcode-sequencing screen after amphotericin B treatment.

Biological Function	Growth Modes for Mutant Identification		
	Biofilm	Planktonic	Biofilm+Planktonic
Lipid metabolism	*tgl1 cki1*	*ncp1 inp51 ura7 ecm22*	*sac1 erg5 cyb5 tlg2*
Translation	*rpp1b ssz1*	*rps22b rpl40b rpl42a rps17b rpl6b rpl24a*	*rpl8b rps21a*
Protein metabolism	*hrt3 mnn4*	*rpn10 ste14 glr1 ubc4 ptc7*	*eug1 doa4 jem1*
RNA metabolism	*brr1 pet127 isu1 ngl2*	*set2 ssn2 sas2 gbp2 syf2 ski2 hbs1 ynr024w sfl1 tea1 vts1 nut1 edc3 pih1 pnc1 nsr1 trm11 yll029w nam7*	*ssn3*
Cell cycle	*ptc2 bud9 ndt80 mrc1 gic1 hsl7*	*rme1 msc7 far8 pcl9 ama1*	*elm1 gin4*
Intracellular transport	*shr5 ypt7 pep3 age2*	*srp101 aps3 pex21 tom5 jjj1 fmp37 vtc1 fmp43 btn2 vps13 arx1 yer128w xdj1*	*osh2 vta1 apm3*
Small molecule metabolism	*ypl033c*	*trp4 bna2 gcv1*	*sah1 ykl151c*
TORC1 signaling	*gtr2*		*gtr1 meh1 slm4 slm1*
Other functions	*gal83 mdh2 pck1 sak1 ynr064c srl2 pbp2 YBL055C*	*cis3 spo71 dit2 qcr8 rip1 yjl055w inp1 ylr073c pgm2 ymr099c ynl191w mms2 gyp1 arn1 sam3*	*ras2 sma2 ybr238c rho3 izh3 vht1 zrt1*
Unknown	*ylr283w om45 dlt1 ylr064w ylr278c ybr255w ypl068c*	*ecm27 pib2 ynl208w yal049c ylr125w rmd8 met12 mpa43 ytp1 ymr181c ylr407w ygr259c yor292c nrp1 ybr016w yor139c ybl081w tma16*	*asg1 ygr182c ygr190c yhr159w*

Mutants significantly more abundant in mixed populations of knockout mutants after treatment with amphotericin B in biofilm and planktonic populations. Mutants are classified according to the gene ontology of the corresponding wild type alleles by biological processes. See Supplementary Table S2 for more details.
