# Supplementary material for: A common mechanism involving the TORC1 pathway can lead to amphotericin B-persistence in biofilm and planktonic Saccharomyces cerevisiae populations
Source: Sci Rep. 2016 Feb 23;6:21874. doi: 10.1038/srep21874 (PMC4763212; doi:10.1038/srep21874)
Supplement: Supplementary Information [file srep21874-s1.pdf]

SUPPLEMENTARY INFORMATION

A common mechanism involving the TORC1 pathway can lead to amphotericin B-persistence in biofilm and planktonic *Saccharomyces cerevisiae* populations

Rasmus Bojsen, Birgitte Regenberg, David Gresham, and Anders Folkeson

Supplementary Figure S1. Pooled haploid gene deletion mutants were cultivated as biofilm or planktonic cells in four biological replicates (colored circles). After four days, half of the populations were treated with 10 µg/ml amphotericin B (AmB) for 12 hours (+ AmB) or untreated (- AmB) as controls. Surviving cells were enriched by outgrowth in YPD before barcode sequencing.

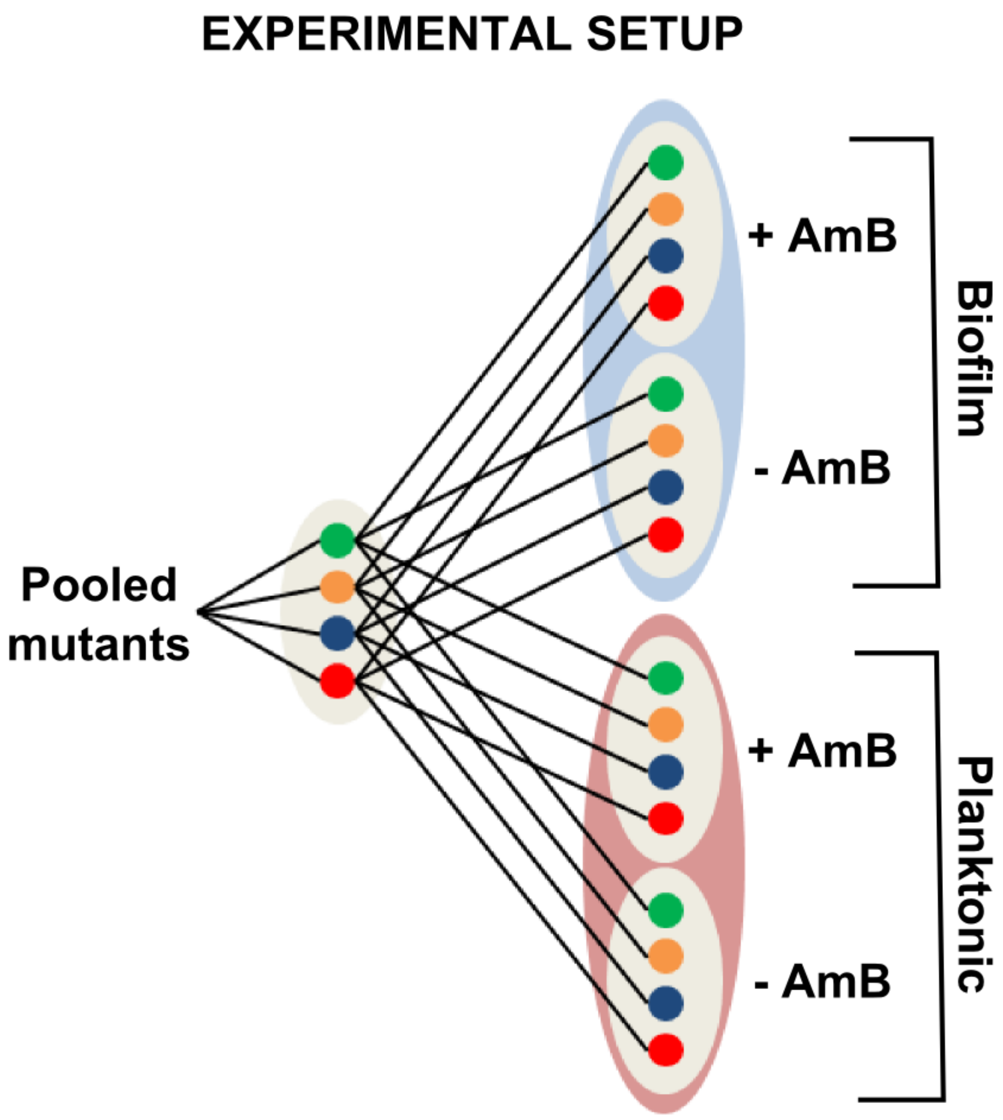

Supplementary Figure S2. Multidimensional scaling plot show reproducibility of the four biological replicates. Untreated biofilm cells (blue circles), untreated planktonic cells (red circles), amphotericin B (AmB)-treated biofilm cells (blue triangles), and AmB-treated planktonic cells (red triangles, two of them are on top of each other in the lower right corner). Distances correspond to leading log-fold-changes between each pair of samples.

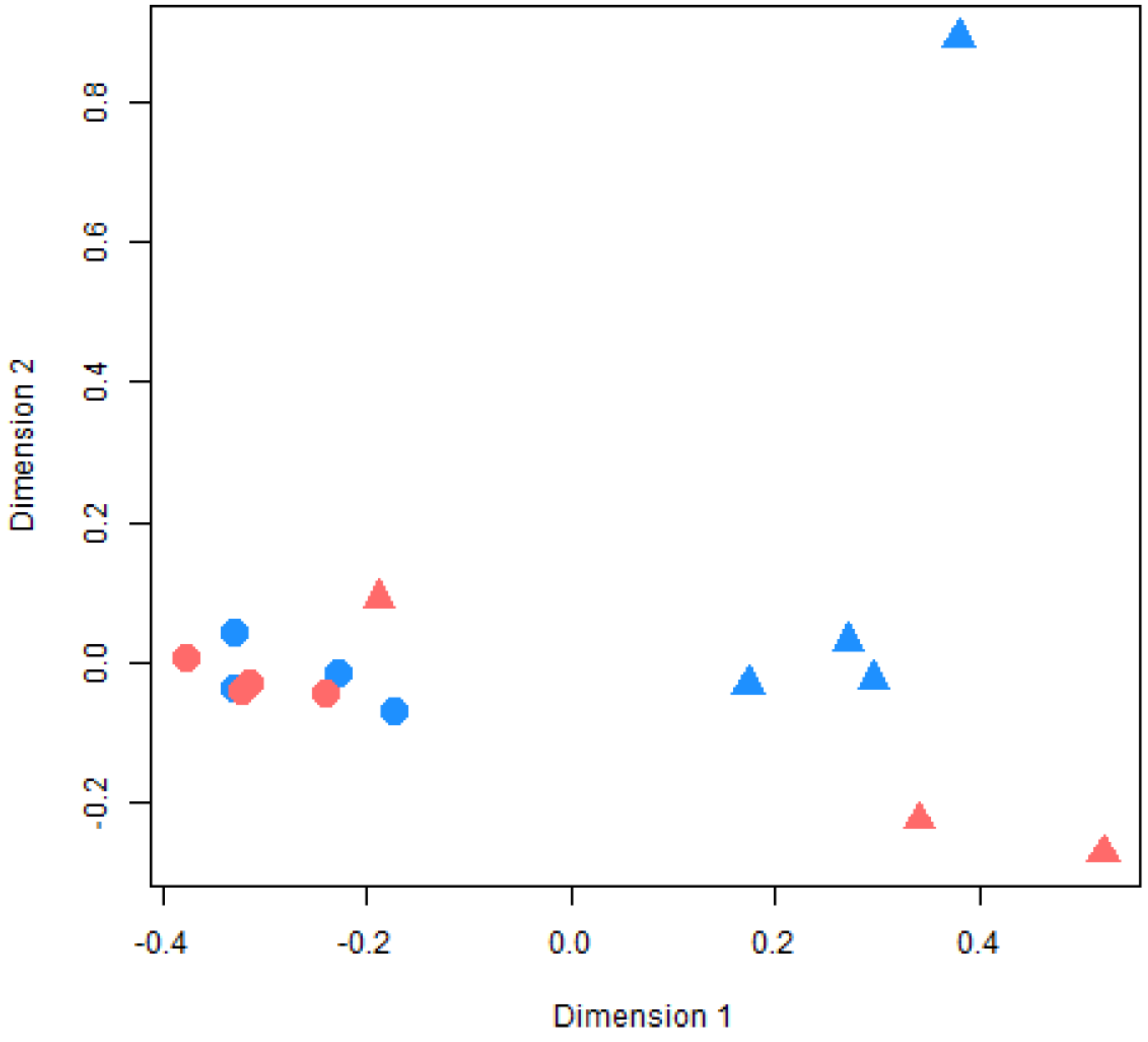

Supplementary Figure S3. Log_{10} fold change (logFC) in sequencing reads between treated and untreated samples and total abundance of mutants in mixed populations. Measurements are log_{10} counts per million (logCPM). (**a**) Smear plot of correlation between mutant abundancy after amphotericin B (AmB) treatment and total number of sequencing counts for biofilm-grown cells. (**b**) As (a) for planktonically grown cells.

a **b**

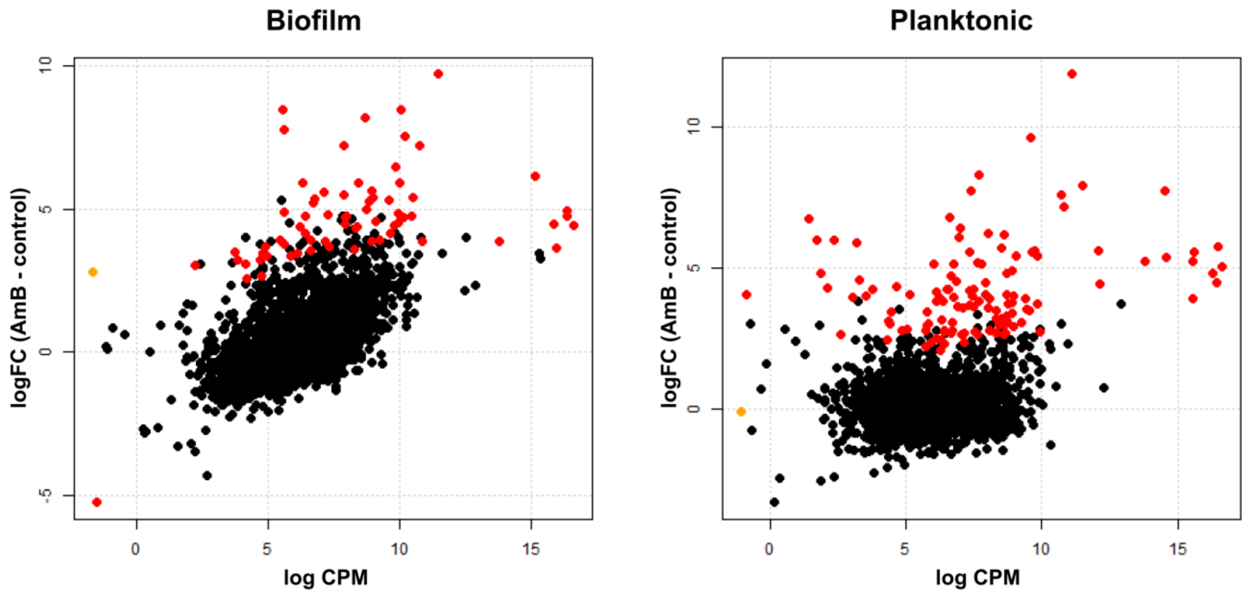

Supplemenatry Figure S4. Log number of sequencing reads of uptag (UP) and downtag (DOWN) barcodes.

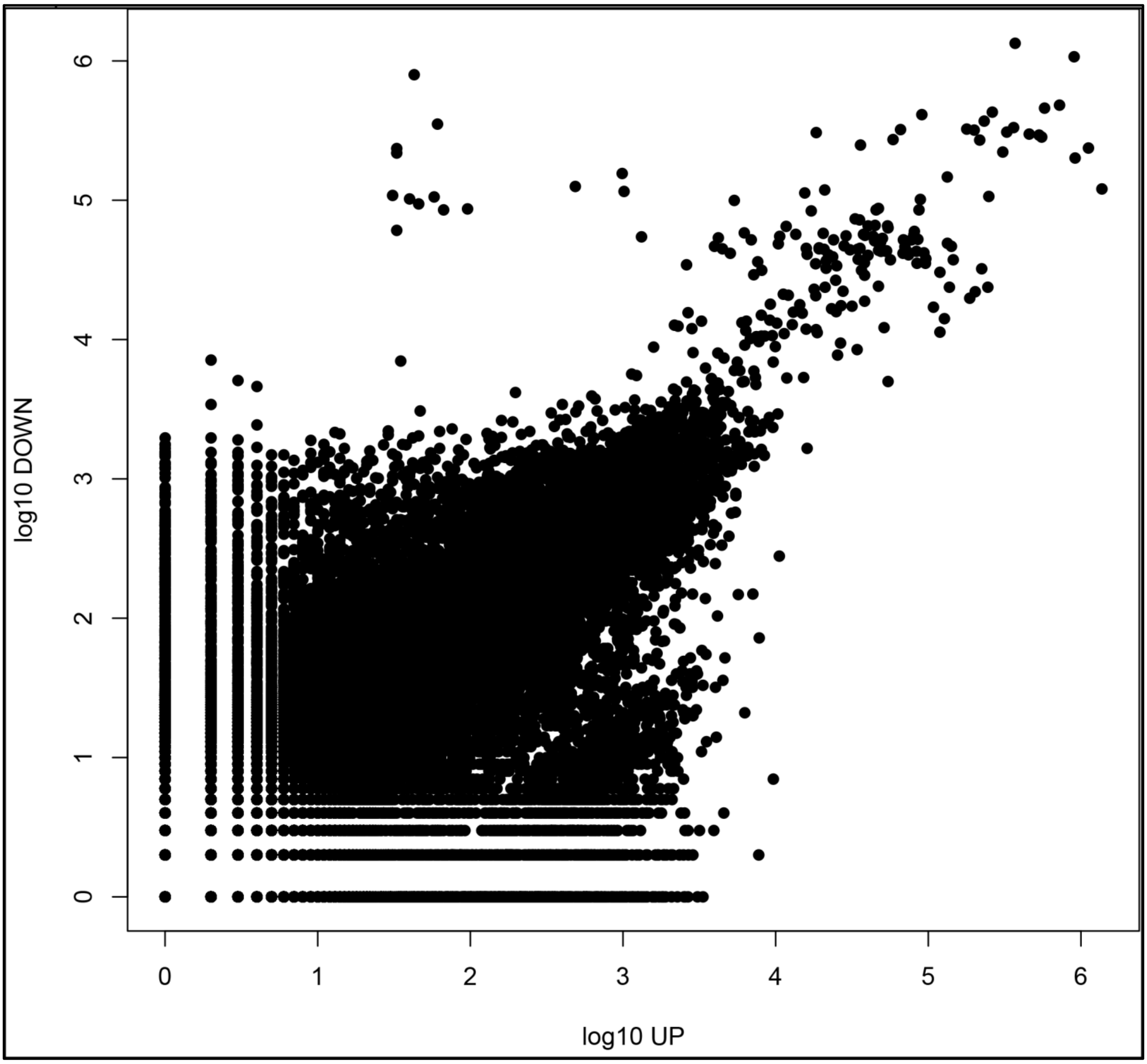

Supplementary Figure S5. Comparison of mean barcode count with associated variance. Red X, average variance per bin; blue point, estimated variance of each mutant after dispersion shrinkage.

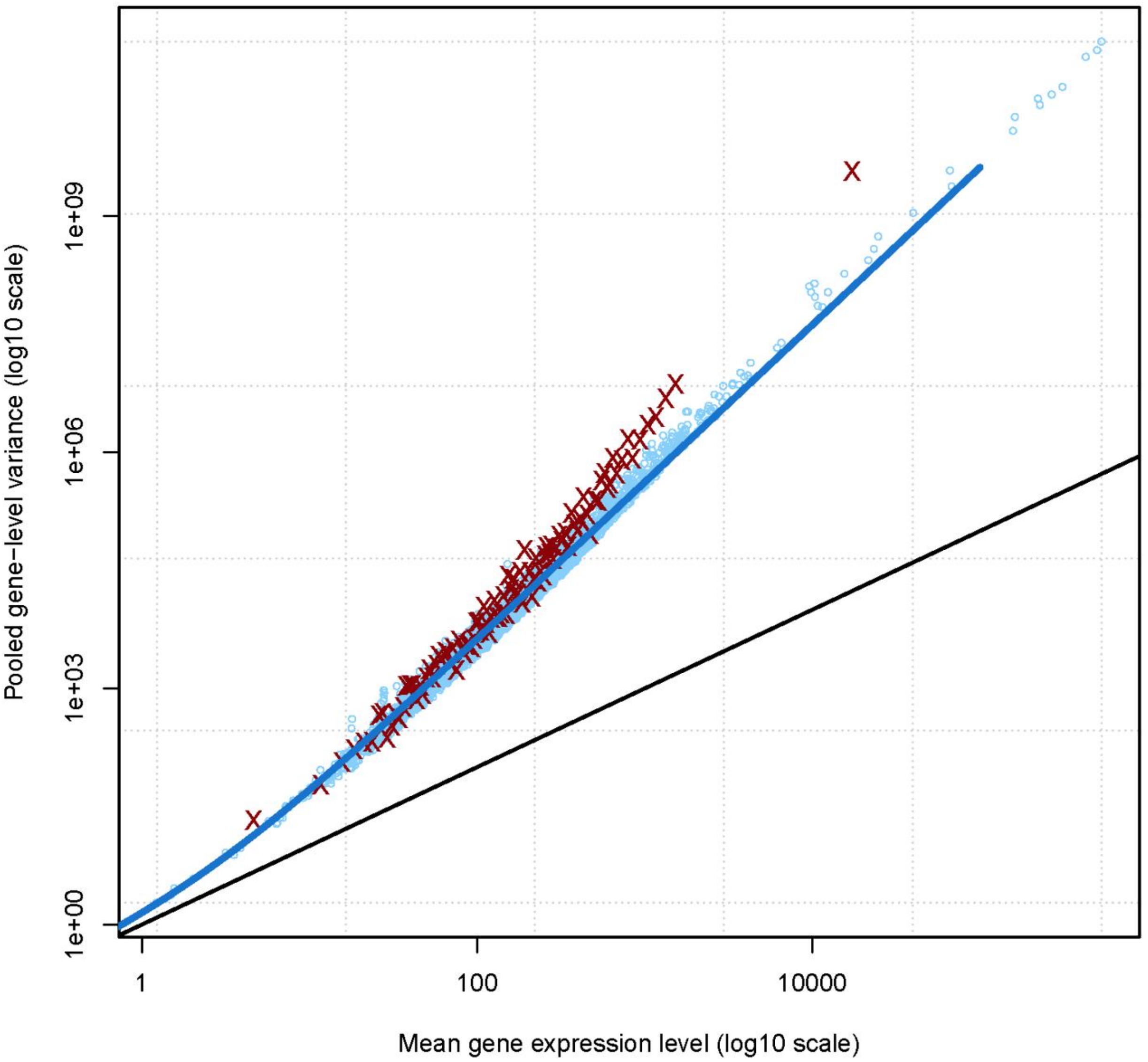

Supplementary Table S1. Fold-change in mutant abundance before and after amphotericin B treatment

Supplementary Table S2. Significantly enriched mutants after amphotericin B treatment grouped by annotated function

Supplementary Table S3. Amino acid concentrations used in minimal medium.

**Supplement**	**Medium concentration (mg/l)**
L-Adenine	35
L-Alanine	120
L-Arginine	120
L-Aspargine	120
L-Aspartic acid	120
L-Cysteine	180
L-Glutamic acid	180
L-Glutamine	120
Glycine	120
L-Histidine	120
L-Isoleucine	180
L-Leucine	180
L-Lysine	180
L-Methionine	75
L-Phenylalanine	75
L-Proline	120
L-Serine	120
L-Threonine	150
L-Tryptophan	120
L-Tyrosine	45
L-Valine	180
Uracil	120

Supplementary Table S4. Primers used for barcode sequencing[1].

Primer name	Primer sequence
Illumina UPTAG Index 1	ACG CTC TTC CGA TCT ATACC GTC CAC GAG GTC TCT
Illumina DNTAG Index 1	ACG CTC TTC CGA TCT ATACC GTG TCG GTC TCG TAG
Illumina UPTAG Index 19	ACG CTC TTC CGA TCT GGAAC GTC CAC GAG GTC TCT
Illumina DNTAG Index 19	ACG CTC TTC CGA TCT GGAAC GTG TCG GTC TCG TAG
Illumina UPTAG Index 20	ACG CTC TTC CGA TCT CTCAG GTC CAC GAG GTC TCT
Illumina DNTAG Index 20	ACG CTC TTC CGA TCT CTCAG GTG TCG GTC TCG TAG
Illumina UPTAG Index 21	ACG CTC TTC CGA TCT ACTGG GTC CAC GAG GTC TCT
Illumina DNTAG Index 21	ACG CTC TTC CGA TCT ACTGG GTG TCG GTC TCG TAG
Illumina UPTAG Index 22	ACG CTC TTC CGA TCT TACAT GTC CAC GAG GTC TCT
Illumina DNTAG Index 22	ACG CTC TTC CGA TCT TACAT GTG TCG GTC TCG TAG
Illumina UPTAG Index 23	ACG CTC TTC CGA TCT GGCAT GTC CAC GAG GTC TCT
Illumina DNTAG Index 23	ACG CTC TTC CGA TCT GGCAT GTG TCG GTC TCG TAG
Illumina UPTAG Index 24	ACG CTC TTC CGA TCT CAGTT GTC CAC GAG GTC TCT
Illumina DNTAG Index 24	ACG CTC TTC CGA TCT CAGTT GTG TCG GTC TCG TAG
Illumina UPTAG Index 25	ACG CTC TTC CGA TCT ATACC GTC CAC GAG GTC TCT
Illumina DNTAG Index 25	ACG CTC TTC CGA TCT ACCAG GTG TCG GTC TCG TAG
Illumina UPTAG Index 26	ACG CTC TTC CGA TCT TTAGC GTC CAC GAG GTC TCT
Illumina DNTAG Index 26	ACG CTC TTC CGA TCT TTAGC GTG TCG GTC TCG TAG
Illumina UPTAG Index 27	ACG CTC TTC CGA TCT GCCAC GTC CAC GAG GTC TCT
Illumina DNTAG Index 27	ACG CTC TTC CGA TCT GCCAC GTG TCG GTC TCG TAG
Illumina UPTAG Index 28	ACG CTC TTC CGA TCT CGACC GTC CAC GAG GTC TCT
Illumina DNTAG Index 28	ACG CTC TTC CGA TCT CGACC GTG TCG GTC TCG TAG
Illumina UPTAG Index 70	ACG CTC TTC CGA TCT TGGTG GTC CAC GAG GTC TCT
Illumina DNTAG Index 70	ACG CTC TTC CGA TCT TGGTG GTG TCG GTC TCG TAG
Illumina UPTAG Index 71	ACG CTC TTC CGA TCT GTCCT GTC CAC GAG GTC TCT
Illumina DNTAG Index 71	ACG CTC TTC CGA TCT GTCCT GTG TCG GTC TCG TAG
Illumina UPTAG Index 72	ACG CTC TTC CGA TCT CCTTA GTC CAC GAG GTC TCT
Illumina DNTAG Index 72	ACG CTC TTC CGA TCT CCTTA GTG TCG GTC TCG TAG
Illumina UPTAG Index 95	ACG CTC TTC CGA TCT GCATA GTC CAC GAG GTC TCT
Illumina DNTAG Index 95	ACG CTC TTC CGA TCT GCATA GTG TCG GTC TCG TAG
Illumina UPTAG Index 96	ACG CTC TTC CGA TCT CCGTC GTC CAC GAG GTC TCT
Illumina DNTAG Index 96	ACG CTC TTC CGA TCT CCGTC GTG TCG GTC TCG TAG
Illumina UPkanMX	CAA GCA GAA GAC GGC ATA CGA GAT GTC GAC CTG CAG CGT ACG
IlluminaDNkanMX	CAA GCA GAA GAC GGC ATA CGA GAT ACG AGC TCG AAT TCA TCG
Illumina P5	A ATG ATA CGG CGA CCA CCG AGA TCT ACA CTC TTT CCC TAC ACG ACG CTC TTC CGA TCT

REFERENCES

1. Robinson, D.G., Chen, W., Storey, J.D. & Gresham, D. Design and analysis of Bar-seq experiments. *G3 (Bethesda)* **4**, 11-18 (2014).
